# Evaluation of inter- and intrafractional motion of liver tumors using interstitial markers and implantable electromagnetic radiotransmitters in the context of image-guided radiotherapy (IGRT) – the ESMERALDA trial

**DOI:** 10.1186/s13014-015-0456-y

**Published:** 2015-07-14

**Authors:** Daniel Habermehl, Patrick Naumann, Rolf Bendl, Uwe Oelfke, Simeon Nill, Jürgen Debus, Stephanie E. Combs

**Affiliations:** Department of Radiation Oncology, University Hospital of Heidelberg, Im Neuenheimer Feld 400, 69120 Heidelberg, Germany; Department of Medical Physics in Radiation Oncology, German Cancer Research Center (DKFZ), Heidelberg, Germany; Medical Informatics, Heilbronn University, Heilbronn, Germany; Joint Department of Physics at The Institute of Cancer Research and The Royal Marsden NHS Foundation Trust, London, UK; Department of Radiation Oncology, Klinikum rechts der Isar, Technische Universität München, Ismaninger Str. 22, 81675 Munich, Germany

**Keywords:** Image-guided radiotherapy, IGRT, Fiducial marker, Fiducials, Organ motion management, Organ motion

## Abstract

**Background:**

With the development of more conformal and precise radiation techniques such as Intensity-Modulated Radiotherapy (IMRT), Stereotactic Body Radiotherapy (SBRT) and Image-Guided Radiotherapy (IGRT), patients with hepatic tumors could be treated with high local doses by sparing normal liver tissue. However, frequently occurring large HCC tumors are still a dosimetric challenge in spite of modern high sophisticated RT modalities. This interventional clinical study has been set up to evaluate the value of different fiducial markers, and to use the modern imaging methods for further treatment optimization using physical and informatics approaches.

**Methods and design:**

Surgically implanted radioopaque or electromagnetic markers are used to detect tumor local-ization during radiotherapy. The required markers for targeting and observation during RT can be implanted in a previously defined optimal position during the oncologically indicated operation. If there is no indication for a surgical resection or open biopsy, markers may be inserted into the liver or tumor tissue by using ultrasound-guidance. Primary study aim is the detection of the patients´ anatomy at the time of RT by observation of the marker position during the indicated irradiation (IGRT). Secondary study aims comprise detection and recording of 3D liver and tumor motion during RT. Furthermore, the study will help to develop technical strategies and mechanisms based on the recorded information on organ motion to avoid inaccurate dose application resulting from fast organ motion and deformation.

**Discussion:**

This is an open monocentric non-randomized, prospective study for the evaluation of organ motion using interstitial markers or implantable radiotransmitter. The trial will evaluate the full potential of different fiducial markers to further optimize treatment of moving targets, with a special focus on liver lesions.

## Background

Primary liver tumors (PLC) represent a great challenge in radiooncology because tumor sizes are often large and represent a relatively high proportion of liver tissue [1]. Furthermore, high radiation doses are needed for long-term control of primary liver cancer. Incidence of hepatocellular carcinoma (HCC) is raising in western countries mostly due to increasing hepatitis c infections. Current therapeutic approaches for HCC and other PLC are complete surgical resection, liver transplantation and locoregional ablative therapies including radiofrequency ablation and chemoembolization. In case of locally advanced or metastasized tumors the multi-kinase inhibitor Sorafenib (Nexavar®) has proven efficacy and leads to prolonged overall survival compared to placebo [2].

Radiation therapy has failed to show promising results in the past because conventional photon techniques lead to a high dose deposition in the normal liver which can potentially cause Radiation-Induced Liver Disease (RILD) [3]. With the development of more conformal and precise radiation techniques such as Intensity-Modulated Radiotherapy (IMRT), Stereotactic Body Radiotherapy (SBRT) and Image-Guided Radiotherapy (IGRT) over the last two decades, patients with hepatic tumors (mainly not suitable for standard therapies) could be treated with high local doses by sparing normal liver tissue and showed good short- and long-term responses [4–7]. However, frequently occurring large HCC tumors are still a dosimetric challenge in spite of modern high sophisticated RT modalities due to the limited hepatic tolerance and limited hepatic function in this patient subgroup with a high frequency of liver cirrhosis [4, 8].

Treatment of PLC and liver metastases is an interdisciplinary challenge for all involved clinical disciplines. While multiple liver metastases are treated with systemic therapies, oligometastatic patients can undergo local ablative treatment approaches, e. g. surgical resection, RFA and radiotherapy (RT) [5, 9]. Every local ablative approach has pros and cons and clinical decision finding depends on tumor localization, size, proximity to greater vessels and multifocality. In many cases different therapy modalities have to be combined to achieve optimal results. Radiotherapy may play a more important role in centrally located tumors near the porta hepatica, in case of close proximity to the portal or hepatic vein, local relapses or medically inoperable patients.

There are numerous studies on the characterization of respiration-induced tumor motion [10–13]. Attempts to detect tumor motion through the analysis of easily observable surrogate signals (e. g. Anzai-belt, lung volume) finally showed a relatively high grade of uncertainty. Several feasibility studies focused on fiducial- or image-based recognition of organ and tumor motion [14, 15]. These publications showed that the use of radioopaque fiducial markers can successfully minimize margins, increase dose to tumor volume and finally improve clinical results [16].

Dynamic adaptation of dose application due to detection of real-time organ motion requires the prediction of organ motion during time latency of the individual system. A further unsolved problem is the prediction of organs at risk (OARs) during therapy. To compensate for inaccurate irradiation due to organ motion during therapy with a conventional linear accelerator (LINAC) mainly two strategies are possible: adaptation of tumor position through dynamic couch shifting and automatic adaptation of the multi-leaf collimator (MLC). None of these strategies has been successfully integrated in clinical routine. Besides the prediction of respiratory-induced motion another unresolved issue is the secure integration of a real-time motion sensor with an automatic, dynamic MLC conformation into one system.

Surgically implanted radioopaque or electromagnetic markers are used to detect tumor localization during radiotherapy. Radioopaque markers are commercially available and approved as medical product. The electromagnetic markers of the CALYPSO system in the U.S. are approved by the Federal Drug Administration (FDA). The surgical implantation follows a preceding interdisciplinary discussion for the definition of an oncological therapy concept with the aim of extracting a biopsy or a surgical resection of the tumor in one session. The required markers for targeting and observation during RT can be implanted in a previously defined optimal position during the oncologically indicated operation. If there is no indication for a surgical resection or biopsy (open biopsy), markers may be inserted into the liver/tumor tissue by using ultrasound-guidance.

Thus, the present study concept has been set up to evaluate the value of different fiducial markers, and to use the aquired imaging for further treatment optimization using physical and informatics approaches.

## Methods and design

### Primary and secondary study aims

Primary study aim is the detection of the patients´ anatomy at the time of RT by observation of the marker position during the oncologically indicated irradiation (IGRT).

#### Secondary study aims

- Detection and recording of 3D organ motion of the liver during RT
- Development of technical strategies and mechanisms based on the recorded information on organ motion to avoid inaccurate dose application resulting from fast organ motion and deformation

### Study design

The study is a prospective, single-armed, non-randomized clinical trial.

### Therapeutic advantages for patients included in the clinical trial and treatment

The immediate advantage for all patients who will participate in the present trial consists in the procedure of marker implantation for the detection of organ motion before radiotherapy. The radioopaque marker material allows correlation of IGRT generated kV- or MV-based cone-beam-CTs with pretherapeutic treatment planning CT scans and thus provides additional information that improves the accuracy of patient positioning and dose application. For this purpose medically approved interstitial fiducial markers (CE labeled) are applied for clinical use, and furthermore the electromagnetic CALYPSO marker system has FDA approval for comparable indications.

For local ablative treatments a three to eight fraction regimen, e.g. 3×20 Gy (primarily liver tumors) or 8×7.5 Gy(centrally located lung tumors) prescribed to the 65- or 80 %-isodose will be performed. Conventional doses are not planned for patients included in the ESMERALDA-trial. Currently, patients are immobilized using a customized vacuum pillow and an abdominal compression. However, if patients don´t tolerate the compression a free-breathing radiotherapy will be performed, e.g. with a gating technique. Treatment planning will be performed using a 4D-CT scan and free breathing; breath-hold techniques are not establied at our institution. Fiducial marker (except the Calypso system) motion will be analyzed by information derived from the 4D computed tomography of the treatment planning imaging, fluoroscopic imaging in the treatment position/setup and the sequential and interfractional kV-CBCT imaging.

### Toxicity and additional radiation exposure for patients

Implanted markers are approved for clinical use in this setting. There will be no toxicities that exceed the known toxicities resulting from the implantation procedure. In the context of IGRT portal images will be taken regularly and no additional radiation exposure will occur. Through the observation of the marker position and the installation of the monitor system (e.g. Calypso system), there will be an additional time effort of about 15-20 minutes for the patient.

### Study design

This is an open monocentric non-randomized, prospective study for the evaluation of organ motion using interstitial markers or implantable radiotransmitter for the treatment of liver tumors.

### Randomization

None.

### Inclusion criteria

- Indication for high precision radiotherapy of primary and secondary liver tumors using IGRT
- Age ≥ 18 years of age
- ability of subject to understand character and individual consequences of the clinical trial
- written informed consent (must be available before enrolment in the trial)

### Exclusion criteria

- refusal of the patients to take part in the study
- medical reasons impeding marker implantation or IGRT for treatment of liver tumors.
- non-compliance of patients

### Study plan and duration of regular study participation

1. Interdisciplinary consent for the indication of high precision RT of primary or secondary liver tumors
2. Information about the ESMERALDA study with a focus on trial sequence, risks and adverse events
3. Submission of the written informed consent
4. Implantation of the required fiducial markers for IGRT in the context of an oncologically indicated operation or during an ultrasound-guided procedure
5. Imaging studies for radiotherapy treatment planning
6. Radiotherapy treatment planning
7. Radiotherapy with daily IGRT and/or the CALYPSO system
8. Planned study end: 6-8 weeks after the end of RT (first oncolgical follow-up)

Acquired data will be pseudonymised for further technical examinations and computations.

### Concomitant therapies

n/a

### Study duration

The recruiting phase of the study will end after inclusion of the planned patient number of *n* = 50.

#### Premature study end

New scientific findings may lead to a premature end of the study. Decision on study closing will be made by all participating persons.

### Statistical design and analysis

This prospective, non-randomized clinical trial is planned to include 50 patients with primary (*n* = 25) or secondary (*n* = 25) liver tumors. The proposed number of patients is an approximated estimation for a sufficient investigation of parameters in both groups which allows a finalization during the mentioned timeline. A dedicated power calculation is not adequate and was therefore not performed. Motion data of the initial 4D treatment planning CT will be correlated with on-board fluoroscopic and CBCT-based motion and calculated shift vectors. Intrafraction as well as interfraction motion will be measured. Furthermore, putative fiducial migration will be analyzed. In summary, the study will help to establish a motion-management strategy, also including the significance of the number of markers and their spatial correlation.

### Ethical and legal aspects

#### Declaration of helsinki

The study is in compliance with the Declaration of Helsinki (2008 Version of the Declaration of Helsinki, adopted at the 59th WMA General Assembly, Seoul, October 2008).

#### Ethical aspects

The study protocol as well as the patients´ informed consent and the patient information were submitted to the Ethical Commission of the University of Heidelberg for appraisal. The assigned in-house number is S-112/2012. All changes of the study protocol, especially those concerning patient safety, will be communicated to the study committee of the Ethical Commission.

#### Patients´ informed consent

Only those patients who agreed to participate in the study after detailed oral and written information will be included. Study participation is voluntary and can be withdrawn at any time by the patients´ choice without any reasons.

#### Funding

This study protocol has not undergone peer-review by a funding body.

#### Study registration

The study is registered on clinicaltrials.gov with the following ID: NCT02095236.

#### Status of the study

The study is currently recruiting patients.

## Discussion

From a radiation oncology perspective, optimization of dose distributions as well as motion management in moving targets is of utmost priority. For the latter, motion surrogates can be implemented, such as fiducial markers placed into the tumor or into its surrounding tissue. The present one-armed non-randomized trial will evaluate the full potential of different fiducial markers to further optimize treatment of moving targets, with special focus on liver lesions.
